# Intraperitoneal Injection of Human Ferritin Heavy Chain Attenuates the Atherosclerotic Process in APOE-Knockout Mice

**DOI:** 10.3390/jcdd10070309

**Published:** 2023-07-19

**Authors:** Wanzhong Yuan, Jianlin Zhang, Ran Huo, Chaofan Hou, Jun Yang, Tao Wang

**Affiliations:** 1 Department of Neurosurgery, Peking University Third Hospital, Beijing 100191, Chinahcfky@outlook.com (C.H.);; 2 Center of Basic Medical Research, Institute of Medical Innovation and Research, Peking University Third Hospital, Beijing 100191, China; 3 Department of Radiology, Peking University Third Hospital, Beijing 100191, China

**Keywords:** ferritin heavy chain, atherosclerosis, transferrin receptor, iron, APOE-knockout mice

## Abstract

**Background:** Iron overload can accelerate the accumulation of lipid oxides and contribute to the progression of atherosclerosis. Ferritin heavy chain (FT-H) exhibits oxidase activity, which inhibits the toxicity of ferrous ions and reduces oxidative damage. We investigated the effect of the intraperitoneal injection of FT-H on the progression of atherosclerosis in APOE-knockout mice (Apo-E^(−/−)^ mice). **Methods:** All mice were fed on a high-fat diet. After 10 weeks, the mice were divided into an injection group (*n* = 4) and a control group (*n* = 4). The injection group was injected intraperitoneally with FT-H (50 mg/kg, once a week), and the control group was treated with PBS buffer (at an equal volume to the injection group, once a week). After 10 weeks of intervention, MRI of the aortas was performed. Then, the animals were sacrificed, and tissues were taken. Hematoxylin–eosin (HE) staining was used for histomorphometry, Masson staining was used to quantify the collagen content in the arteries, Prussian blue staining was used to visualize iron deposition in the arteries, and MRI was used to analyze the structure of the aorta in vivo. Immunohistochemistry was performed to detect the expression of MCP-1, MMP-2, MMP-9, FT-H, FT-L, TfR1, NRF-2 and GPX-4. **Results:** The serological results showed that the injection group had lower levels of glucose (Glu), triacylglycerol (TG), cholesterol (CHO), low-density lipoprotein-C (LDL-C) and malondialdehyde (MDA) (*p* = 0.0058, *p* = 0.0098, *p* = 0.0019, *p* = 0.0368 and *p* = 0.0025, respectively), and their serum ferritin (SF) and superoxide dismutase (SOD) levels were higher (*p* = 0.0004 and *p* < 0.0001). The Masson staining and MRI results showed that the injection group had less collagen deposition (*p* = 0.0226), a larger arterial lumen area and arterial volume (*p* = 0.0006 and *p* = 0.0005), thinner arterial wall thickness (*p* = 0.0013) and a more stable arterial plaque structure (*p* < 0.0001). The immunohistochemical results showed reduced expression of FT-H, FT-L, TfR1, MMP-2, MMP-9, MCP-1 and NRF-2 in the injection group (*p* = 0.0054, *p* = 0.0242, *p* = 0.0221, *p* = 0.0477, *p* = 0.0131, *p* = 0.0435 and *p* = 0.0179). Prussian blue staining showed that the area of iron-positive areas in the aortic plaques of the control group was larger than that of injected group. The expression of GPX-4 was lower in the control group than in the injection group (*p* = 0.016). **Conclusions:** The intraperitoneal administration of FT-H to Apo-E^(−/−)^ mice resulted in lower blood glucose and lipid levels; reduced iron and iron metabolism protein deposition in the aorta; reduced indices of their ferroptosis, oxidation and inflammatory aggregation; and reduced collagen deposition in the aorta, which delayed the process of aortic atherosclerosis in mice.

## 1. Background

Atherosclerotic disease is the most significant threat to global health and a major public health problem that seriously affects people’s livelihoods of in several nations [1]. Atherosclerosis is a systemic inflammatory process that begins in its initial phase with endothelial cell dysfunction, characterized by increased expression of adhesion and inflammatory molecules such as VCAM-1, ICAM-1, MCP-1 and IL-6; subsequently, through collagen deposition and lipid accumulation, atherosclerotic plaques are formed, leading to narrowing of the arterial lumen; in the later stages, metalloproteinases such as MMP-2, MMP-9 and other metalloproteinases, lead to ischemic adverse cardiovascular events by regulating collagen degradation in the extracellular matrix and rupture of the atheromatous plaque [2,3]. In addition, MCP-1 can promote the progression of the atherosclerotic process by mediating the aggregation of inflammatory cells [4].

Iron metabolic processes, although not associated with the initiation of atherosclerosis, can exacerbate the progression of the atherosclerotic process through ferroptosis-mediated lipid over-deposition when iron is overloaded [5,6]. APOE-knockout mice (Apo-E^(−/−)^ mice) are the most commonly used mouse model of atherosclerosis, with the presence of foam cells detectable at approximately 8 weeks and reaching an advanced stage of atherosclerosis after 20 weeks. This time course is greatly accelerated by feeding mice a high-fat diet [7,8]. In previous animal studies, interventions with Apo-E^(−/−)^ mice, often in the form of low-iron diets, iron dextran intraperitoneal injections and deferoxamine intravenous injections, have found that reduced intravascular iron deposition slows the atherosclerotic process and increases plaque stiffness. The findings of these studies suggest that a reduction in intravascular iron deposition may slow the atherosclerotic process and increase plaque stability [9,10,11,12]. In particular, the upregulation of ferritin heavy chain (FT-H) and transferrin receptor (TfR1) in the aorta may be the mechanism by which iron deposition promotes the progression of atherosclerosis [13].

Many proteins are associated with ferroptosis. The expression of Glutathione Peroxidase 4 (GPX-4) is essential for maintaining intracellular lipid homeostasis, preventing lipid accumulation and blocking the iron-dependent non-apoptotic mode of death [14]. Ferritins are a group of iron storage proteins with detoxifying functions that are essential for iron homeostasis and are involved in a wide range of physiological and pathological processes [15]. Ferritins can be divided into FT-H and ferritin light chain (FT-L) depending on the subunit [16]. FT-H is important for inhibiting the toxicity of ferrous ions and reducing oxidative damage because of its iron oxidase activity, while FT-L contributes to the formation of ferritin core structures [17,18]. In addition, TfR1, which is normally expressed on the cell surface and interacts with transferrin to participate in cellular iron uptake, also plays an important role in the exacerbation of atherosclerosis caused by iron overload [13]. Nuclear factor erythroid 2-related factor 2 (NRF-2), as an important endogenous antioxidant protein, can regulate the increased expression of antioxidant proteins such as FT-H and FT-L through its increased expression, thus achieving antioxidant effects [19,20].

In view of the positive effect of FT-H on iron overload in atherosclerosis, this study was the first to observe whether this intervention could have a positive effect on the atherosclerotic process in mice through the long-term intraperitoneal administration of FT-H to Apo-E^(−/−)^ mice. The observations included (1) changes in atherosclerosis-related serum biochemical markers; (2) changes in the aortic vascular structure; and (3) changes in iron metabolism proteins and atherosclerosis-related immunohistochemical markers in mouse arteries.

## 2. Materials and Methods

### 2.1. Preparation and Treatment of FT-H

The HFn-pET-30a (+) plasmid was synthesized using genetic engineering techniques and transformed into E. coli BL21(DE3) cells using an E. coli BL21(DE3) kit (TransGen Biotech, cat. no. CD601-02). The high-purity recombinant human FT-H was prepared as described in previous studies [21]. Endotoxin was removed from the prepared FT-H using Detoxi-Gel™ Endotoxin Removing Gel (Thermo Scientific, cat. no. 20344). FT-H was diluted to 5 mg/mL and stored at 4 °C for intraperitoneal injection to the injection group of mice. The FT-H intraperitoneal injection concentration was 50 mg/kg in this study, which was based on a previous paper that describes a safe concentration of 480 mg/kg for the intravenous administration of drug-laden FT-H to BALB/c mice [22].

### 2.2. Animal Atherosclerosis Modeling Procedure and Postmodeling Treatment

**Figure 1:**
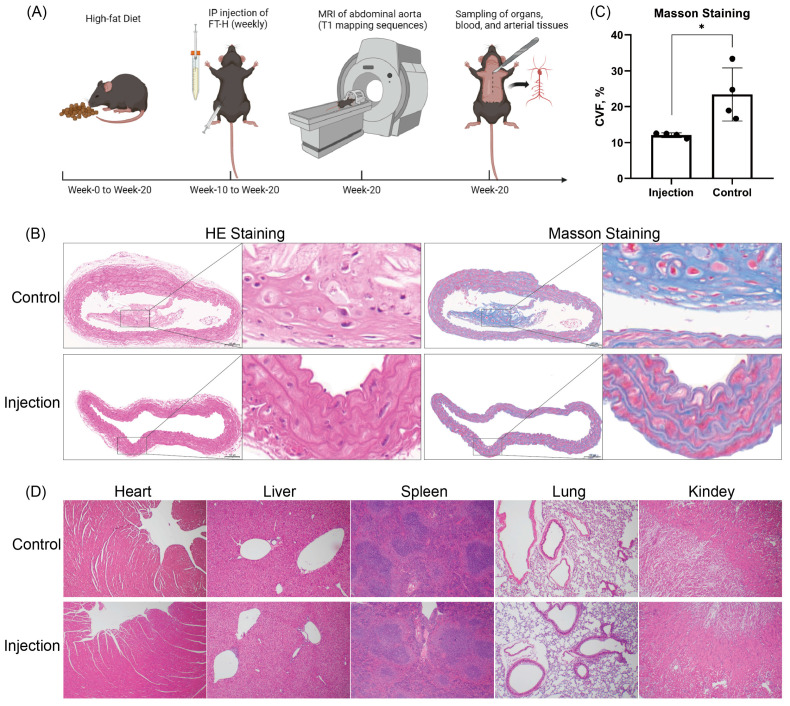
Illustration of the experimental procedure and analysis of HE staining and Masson staining of aortic plaques in Apo-E^(−/−)^ mice. (**A**) Two groups of mice were fed on a high-fat diet for 20 weeks. FT-H was administered intraperitoneally to the injection group at week 10 for 10 weeks. At week 20, mice in both groups underwent abdominal aortic MRI, and tissue and blood sampling was performed at the end of imaging. (**B**) HE staining and Masson staining (10× magnified field of view and 40× magnified field of view) of the aortas of both groups of mice at 20 weeks. Collagen is blue in color and is mainly deposited between the intima and mesentery of the artery. Bar = 100 μm. (**C**) Graph showing the CVF value analysis results from the Masson staining of aortas in the 2 groups of 8 mice. * *p* < 0.05, black dots, visualization of numerical results. (**D**) Images show the HE staining results of the heart, liver, spleen, lungs and kidneys of both groups of mice.

Eight 6- to 8-week-old Apo-E^(−/−)^ mice (four females and four males, with a C57BL/6 genetic background) were purchased from Beijing Vital River Laboratory Animal Technology Co., Ltd (Beijing, China). All animals were obtained in accordance with the Guide for the Care and Use of Laboratory Animals published by the National Institutes of Health. The experiments were approved by the animal care and use committee of the host institution. Mice were housed in a specific pathogen-free (SPF) controlled environment (temperature 22–26 °C, humidity 40–60%, 12 h light/dark cycle). Mice were fed for 20 weeks on a high-fat diet (22.5% by weight; 33.7% of kcal from fat) purchased from BiotechHD Co., Ltd (Beijing, China). At week 10, 8 mice were randomly and equally grouped into an injection group and a control group. The 4 mice in the injection group were injected intraperitoneally once a week with FT-H (5 mg/mL, 200 µL), and the 4 mice in the control group were injected intraperitoneally once a week with PBS buffer (200 µL). The intraperitoneal injection was continued at 50 mg/kg every week thereafter. The intraperitoneal injections were continued for 10 weeks. The experimental procedure is shown in Figure 1A.

### 2.3. MRI and Image Reconstruction of the Abdominal Aorta

MRI of the abdominal aorta in Apo-E^(−/−)^ mice at week 20 was performed using a Bruker BioSpec 9.4T MRI scanner (BioSpec 94/30USR 9.4T Bruker, Ettlingen, Germany) with a mouse head/mouse body coil. Briefly, each mouse was anesthetized with isoflurane (United States Pharmacopeia grade 100%; RWD Life Science, San Diego, CA, USA) in a small container and anesthetized with isoflurane (1–3%) in a 2:1 mixture of air (0.3 L/min) and oxygen (0.1 L/min). The mice were continuously anesthetized with the mixture. The mice were placed in a supine position on a specially designed stand with an adjustable nose cone. A respiratory sensor connected to a monitoring system (SA Instruments, Stony Brook, NY, USA) was placed on the abdomen to monitor respiratory rate and depth. MR scans were performed on the abdominal aortic segments of the mice due to the effects of respiratory movements. Images (11 levels in total) were obtained using localization and T1 sequences. For all MR images, vascular reconstruction was performed by 2 physicians with more than 5 years of experience in cerebral vascular imaging using commercially available software (Vascular Explorer 2, Simulation Medical, Beijing, China). Two observers were unaware of the mouse information. The vessel lumen and vessel wall boundaries were manually outlined on each axial MRI of the index lateral abdominal aorta during the T1 sequence. Plaque burden was measured, including the mean values of the wall area (WA), wall volume (WV), lumen area (LA), vessel area (VA), normalized wall index (NWI = WA/VA × 100%) and maximum wall thickness (Max WT). Among these, NWI is a measurement that uses vessel wall area to standardize plaque size and allows for the comparison of plaques in vessel segments of different sizes, providing a reliable estimate of plaque detection and measurement [23].

### 2.4. Determination of Biochemical Parameters in Serum

The mice were anesthetized with isoflurane (1–3%). Blood samples were taken, and then, the mice were euthanized. The sera obtained after centrifugation (3000× *g* for 15 min at 4 °C) were used to evaluate various serum biochemical parameters. Serum was analyzed for cholesterol (CHO), triglycerides (TG), high-density lipoprotein-C (HDL-C), low-density lipoprotein-C (LDL-C), serum iron (SF), superoxide dismutase (SOD), malondialdehyde (MDA) and glucose (Glu) according to the instructions of the commercial test kits (GM1113, GM1114, GM1115, GM1116, G4301-48T, GM1133, G4300-96T and GM1118, respectively; Servicebio; Wuhan, China). Among them, SOD acts as an antioxidant enzyme and reduces the toxicity of reactive oxygen species to blood vessels [24]. MDA, a lipid peroxide, can greatly impair the anti-atherosclerotic function of HDL [25].

### 2.5. Histopathological Staining

After aortic sampling, a portion was stained with Oil Red O to enable us to qualitatively visualize the distribution of lipids in the aorta and to quantitatively measure lipid levels. The other portion was fixed with 4% paraformaldehyde, paraffin-embedded and sectioned, followed by hematoxylin–eosin (HE) staining to observe the aortic plaque. Masson staining of the specimens was then performed to observe collagen deposition within the arteries, and Prussian blue staining to visualize iron deposition in the arteries. Positive expression of FT-H (anti-FTH rabbit mAb; clone EPR3005Y; 1:300; Abcam; Cambridge, UK), FT-L (anti-FTL mouse pAb; clone 1F9F5; 1:200; Proteintech; Wuhan, China), TfR1 (anti-TfR1 rabbit mAb; clone EPR20584; 1:500; Abcam; Cambridge, UK), MMP-2 (anti-MMP2 rabbit pAb; GB11130; 1:1000; Servicebio; Wuhan, China), MMP-9 (anti-MMP9 mouse mAb; GB12132; 1:500; Servicebio; Wuhan, China), MCP-1 (anti-MCP1 rabbit pAb; GB11199; 1:500; Servicebio; Wuhan, China), NRF-2 (anti-NRF2 rabbit pAb; GB113808; 1:200; Servicebio; Wuhan, China) and GPX-4 (anti-GPX4 1 rabbit pAb; GB114327; 1:1000; Servicebio; Wuhan, China) was observed following immunohistochemistry. All sections were scanned under a Pannoramic Scanner (Pannoramic DESK, P-MIDI, P250; 3D HISTECH; Hungary) and were observed at the same magnification (10×) and with the same viewing parameters. Panoramic images of the observed tissue sections were converted to digital images for immunohistochemical analysis. The optical density of the immune sections correlated with the expression of positively stained proteins, and the average optical density (AOD) was used to analyze the amount of protein expression in each region within the observed sections. The AOD values for different proteins (FT-H, FT-L, TfR1, MMP-2, MMP-9 and MCP-1) were calculated for all sections using ImageJ software (NIH, Bethesda, MD, USA).

### 2.6. Statistical Analysis

Normally distributed continuous variables are expressed as the means and standard deviations, and non-normally distributed variables are expressed as medians and interquartile spacing. Categorical variables are described as counts and percentages. Independent *t* tests and Mann–Whitney U tests were used to determine whether there were significant differences between the two groups of mice in terms of the body weight, serum biochemistry, metabolic parameters, abdominal aortic structure, collagen deposition and AOD values of the different proteins assessed via immunohistochemistry. Statistical analyses were performed using IBM SPSS Statistics 26.0 (SPSS Inc., Chicago, IL, USA). Statistical significance was considered when *p* < 0.05 (two-tailed).

## 3. Results

### 3.1. Intraperitoneal Injection of FT-H for 10 Weeks Improved Collagen Deposition in the Abdominal Aortas of Atherosclerotic Apo-E^(−/−)^ Mice

There were no significant differences in body weight between the two groups of mice at week 0, week 10 and week 20 (*p* = 0.1757, *p* = 0.5020 and *p* = 0.1061, respectively). The injection group showed no adverse effects, such as poor mental health, decreased appetite or illness, during the 10 weeks of FT-H intraperitoneal injection. The Masson staining results of the aorta in both groups are shown in Figure 1B. Collagen deposits were mainly concentrated in the stroma of the subintima of the artery. At week 20, Figure 1C shows that there was a significant difference in the mean collagen volume fraction (CVF) between the injection group and the control group (*p* = 0.023, 12.048 ± 0.639 vs. 23.418 ± 7.435%). At week 20, histological observations did not reveal any significant differences in visceral HE staining in the injection group compared to the control group (Figure 1D).

### 3.2. Intraperitoneal Injection of FT-H for 10 Weeks Improved the Structure of the Abdominal Aorta in Apo-E^(−/−)^ Mice with Atherosclerosis

At week 20, the mean results for the arterial lumen area, arterial lumen volume, arterial vessel wall area, arterial vessel wall volume, arterial wall thickness and NWI in the injection group and the control group were 0.925 ± 0.057 vs. 0.715 ± 0.026 mm^2^, 5.090 ± 0.298 vs. 3.943 ± 0.156 mm^3^, 0.650 ± 0.062 vs. 0.823 ± 0.026 mm^2^, 3.578 ± 0.340 vs. 4.508 ± 0.148 mm^3^, 0.168 ± 0.013 vs. 0.210 ± 0.008 mm and 41.540 ± 1.924 vs. 52.398 ± 1.248%, respectively.

The intraperitoneal injection of FT-H reduced the degree of stenosis in the lumen of the abdominal aorta in atherosclerotic Apo-E^(−/−)^ mice. At week 20, the arterial lumen area and arterial lumen volume were significantly higher in the injection group than in the control group (*p* = 0.0006 and *p* = 0.0005, respectively). The arterial wall thickness, arterial lumen area and arterial lumen volume were significantly lower in the injection group than in the control group (*p* = 0.0013; *p* = 0.0021; and *p* = 0.0024, respectively).

The intraperitoneal injection of FT-H reduced the vulnerability of abdominal aortic plaques during the atherosclerotic process in Apo-E^(−/−)^ mice. At week 20, the NWI of the injection group was significantly lower than that of the control group (*p* < 0.0001).

**Figure 2:**
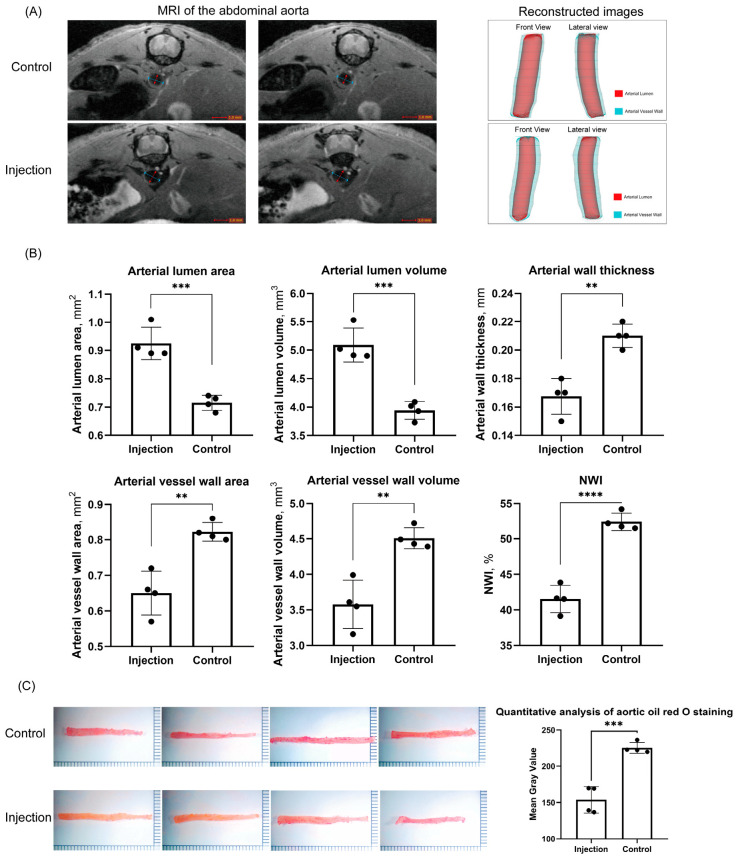
Analysis of aortic plaque and aortic revascularization in 2 groups of mice. (**A**) Magnetic resonance imaging and reconstruction results of the abdominal aortas of mice at 20 weeks. (**B**) Differential analysis of relevant parameters of aortic structure in 2 groups of mice. (**C**) Results of oil-red O staining and analysis of aorta in 2 groups of mice. ** *p* < 0.01, *** *p* < 0.001, **** *p* < 0.0001, black dots, visualization of numerical results.

The Oil Red O staining results of the aorta in both groups are shown in Figure 2C. At week 20, the results show a significant difference in the mean gray value representing lipid levels in the aorta between the injection and control groups (*p* = 0.003, 153.8 ± 9.093 vs. 225.3 ± 3.743).

The above results can be seen in Figure 2.

### 3.3. Intraperitoneal Injection of FT-H for 10 Weeks Improved the Serum Biochemical and Metabolic Parameters in Atherosclerotic Apo-E^(−/−)^ Mice

At week 20, the mean results for Glu, TG, CHO, LDL-C, HDL-C, Fe, MDA and SOD in the injection group and the control group were 8.580 ± 0.385 mmol/L vs. 14.455 ± 2.785 mmol/L, 0.878 ± 0.041 mmol/L vs. 1.100± 0.112 mmol/L, 9.265 ± 0.196 mmol/L vs. 11.090 ± 0.666 mmol/L, 1.325 ± 0.158 mmol/L vs. 1.610 ± 0.143 mmol/L, 0.928 ± 0.021 mmol/L vs. 0.773 ± 0.074 mmol/L, 17.850 ±0.661 ng/mL vs. 5.500 ± 3.366 ng/mL, 2.893 ± 0.408 µIU/mL vs. 3.917 ± 0.062 µIU/mL and 77.152 ± 1.926 U/mL vs. 53.755 ± 2.626 U/mL, respectively.

The intraperitoneal injection of FT-H reduced the levels of blood glucose and slowed the progression of lipid disorder in Apo-E^(−/−)^ mice. The Glu, TG, CHO and LDL-C concentrations in the injection group were significantly lower than those in the control group (*p* = 0.0058, *p* = 0.0098, *p* = 0.0019 and *p* = 0.0368, respectively). The HDL-C levels in the injection group were significantly higher than those in the control group (*p* = 0.0069).

The intraperitoneal injection of FT-H elevated serum ferritin (SF) levels in Apo-E^(−/−)^ mice. SF levels in the injection group were significantly higher than those in the control group (*p* = 0.0004).

The intraperitoneal injection of FT-H reduced peroxidation levels during atherosclerosis progression in Apo-E^(−/−)^ mice. The MDA levels were significantly lower in the injection group than in the control group (*p* = 0.0025), and the SOD levels were significantly higher in the injection group than in the control group (*p* < 0.0001).

**Figure 3:**
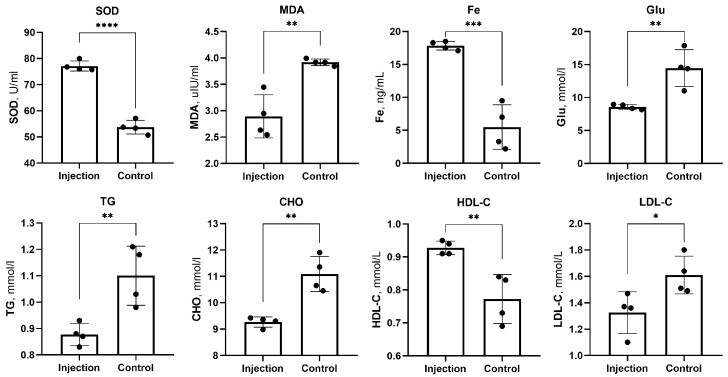
Serum biochemical and metabolic parameters of 8 mice in the 2 groups at 20 weeks. * *p* < 0.05, ** *p* < 0.01, *** *p* < 0.001, **** *p* < 0.0001, black dots, visualization of numerical results.

The above results can be seen in Figure 3.

### 3.4. Intraperitoneal Injection of FT-H for 10 Weeks Improved Iron and Iron Metabolism Protein Deposition and Ferroptosis-Related Protein Expression in the Abdominal Aortas of Atherosclerotic Apo-E^(−/−)^ mice

**Figure 4:**
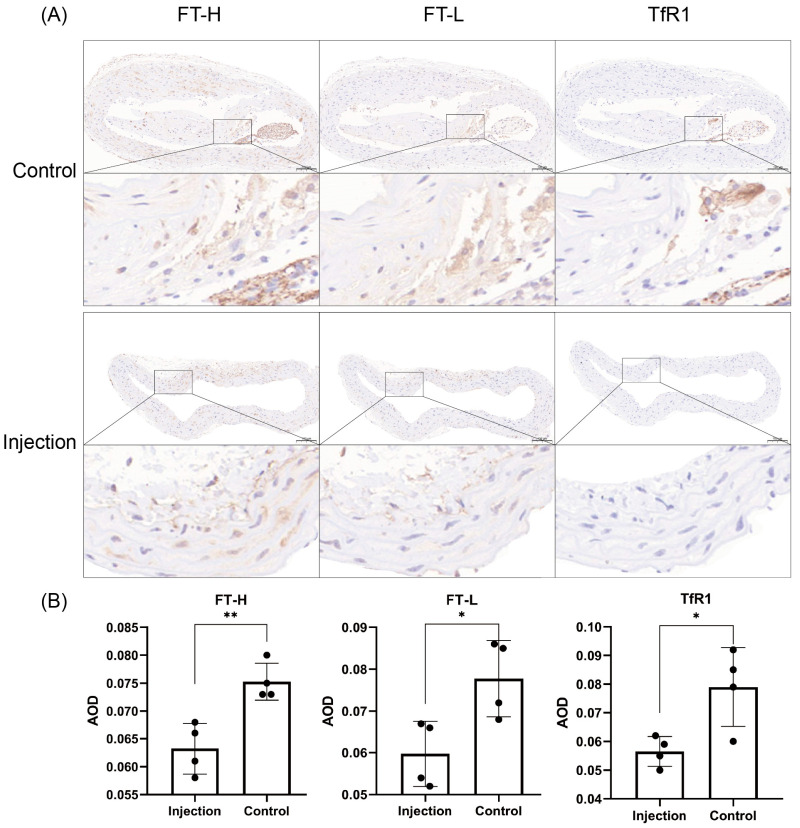
Expression analysis of H-FT, L-FT and TfR1 in the aortas of mice from 2 groups. (**A**) Immunohistochemical results show that the iron metabolism proteins H-FT, L-FT and TfR1 were mainly distributed in the carotid intima and carotid mesentery (*n* = 4). Positive staining was brownish yellow. Bar = 100 μm. (**B**) Graph showing the results of the immunohistochemical AOD value analysis of H-FT, L-FT and TfR1 in the aortas of 8 mice from the 2 groups. * *p* < 0.05, ** *p* < 0.01, black dots, visualization of numerical results.

The mean AODs for FT-H, FT-L and TfR1 in the injection group and the control group at week 20 were 0.063 ± 0.005 vs. 0.075 ± 0.003, 0.060 ± 0.008 vs. 0.078 ± 0.009 and 0.057 ± 0.005 vs. 0.079 ± 0.014, respectively. The immunohistochemistry staining is shown in Figure 4. The deposition of FT-H, FT-L and TfR1 in the aorta was higher in the control group than in the injection group (*p* = 0.0054, *p* = 0.0242 and *p* = 0.0221). FT-H was deposited in the whole artery, and the deposition was greater than that of FT-L and TfR1.

**Figure 5:**
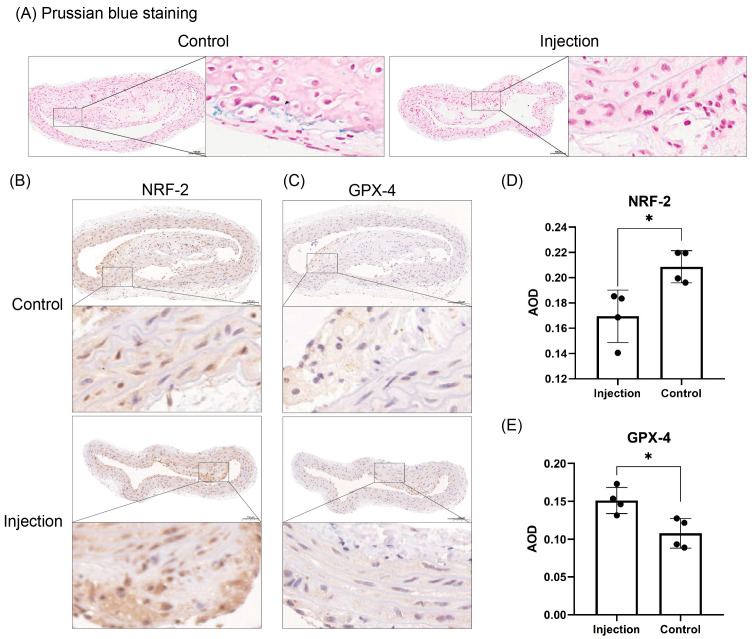
Prussian blue staining of aorta in 2 groups of mice, and analysis of NRF-2 and GPX-4 expression. (**A**) Prussian blue staining showed that the area of iron-positive region in the aortas of mice in the injection group was larger than that in the control group. (**B**–**E**) Immunohistochemical results showing the distribution of NRF-2 and GPX-4 and the results of AOD values in both groups of mice. Positive staining was brownish yellow. Bars = 100 μm. * *p* < 0.05, black dots, visualization of numerical results.

Prussian blue staining showed that the area of iron-positive areas in the aortic plaques of the control group was larger than that of injected group. The expression of NRF-2 was lower and the expression of GPX-4 was higher in the aortas of the injection group (*p* = 0.0179, *p* = 0.0161). The above results can be seen in Figure 5. 

### 3.5. Intraperitoneal FT-H Injection for 10 Weeks Improved Parameters Related to Vulnerability and Inflammatory Chemotaxis of Abdominal Aortic Plaques in Atherosclerotic Apo-E^(−/−)^ Mice

**Figure 6:**
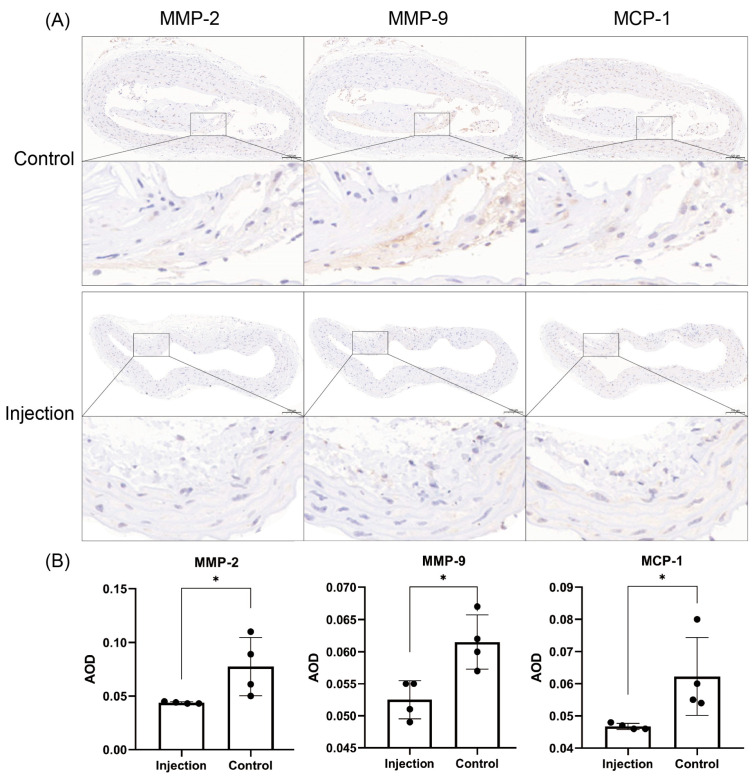
Expression analysis of MMP-2, MMP-9 and MCP-1 in the aortas of mice from the 2 groups. (**A**) Immunohistochemical results show that the iron metabolism proteins MMP-2, MMP-9 and MCP-1 were mainly distributed in the carotid intima and carotid mesentery (*n* = 4). Positive staining was brownish yellow. Bar = 100 μm. (**B**) Graph showing the results of the immunohistochemical AOD analysis of MMP-2, MMP-9 and MCP-1 in the aortas of a total of 8 mice from the 2 groups. * *p* < 0.05, black dots, visualization of numerical results.

The mean AODs for MMP-2, MMP-9 and MCP-1 in the injection group and the control group at week 20 were 0.044 ± 0.001 vs. 0.078 ± 0.027, 0.053 ± 0.003 vs. 0.062 ± 0.004 and 0047 ± 0.001 vs. 0.062 ± 0.012, respectively. The immunohistochemical staining can be seen in Figure 6.

MMP-2 and MMP-9 were lower in the injection group than in the control group (*p* = 0.0477 and *p* = 0.0131, respectively).

Aortic plaque inflammatory tropism was lower in the injection group than in the control group. MCP-1 levels were lower in the injection group than in the control group (*p* = 0.0435).

## 4. Discussion

When iron overload occurs, especially unstable ferrous iron, it can react directly with cells in an oxidative manner, leading to the progression of atherosclerotic disease by producing cytotoxic hydroxyl radicals, which, in turn, promote ferroptosis and accelerate the accumulation of lipid oxides [5,6]. In contrast, ferritin can be regulated by iron overload and oxidative stress, and its main function is to cope with iron overload and reactive oxygen species (ROS) [26]. In previous animal studies, iron metabolism interventions in Apo-E^(−/−)^ mice have been carried out mainly through low-iron dietary feeding, iron dextran intraperitoneal injection and deferoxamine intravenous injection. All of these interventions have been found to reduce iron deposition in Apo-E^(−/−)^ mice, slowing the atherosclerotic process and increasing the stability of plaques. The conclusions of all of these interventions were that the reduction in iron deposition in the blood vessels of Apo-E^(−/−)^ mice slowed the atherosclerotic process and increased the stability of plaques [9,10,11,12]. Due to the anti-iron overload and antioxidant effects of ferritin, our study aims to confirm the effects of this new intervention on serum iron levels, atherosclerosis progression and plaque vulnerability in Apo-E^(−/−)^ mice through the intraperitoneal administration of FT-H.

In Apo-E^(−/−)^ mice, foam cells appear in the blood vessels at approximately 8 weeks [27]. As the local inflammatory response increases, vascular smooth muscle cells begin to migrate into the arterial intima, collagen proliferates at an abnormally high rate and atherosclerotic growth is promoted. Progression to the fibrous atherosclerotic phase occurs after 15–20 weeks, when collagen decreases and plaque vulnerability increases. Our study showed that at week 20, the control group had higher levels of collagen in the subintima of the arteries than the injection group. Previous studies have shown that collagen makes up approximately 60% of the total arterial plaque and can contribute to plaque growth and narrowing of the arterial lumen [28]. This is consistent with our MRI results, where the arterial lumen area and arterial lumen volume of the arteries in the control group were smaller than those in the injection group at week 20; the arterial wall area, arterial wall volume and arterial wall thickness were greater than those in the injection group. The control group had a higher NWI than the injection group, which is a better indicator of plaque volume load and atherosclerosis severity than the degree of stenosis [29,30]. All of these results indicate that the severity of atherosclerosis in the control group was higher than that in the injection group. We speculate that the intraperitoneal injection of FT-H reduced the excessive deposition of collagen in the intima, slowed the arterial lumen stenosis process, and reduced the NWI and arterial plaque volume load. In addition to the structural changes that occurred in the arteries, the immunohistochemical results of this study also showed that MMP-2 and MMP-9 levels were higher in the control group than in the injection group, both of which could increase the vulnerability of atherosclerotic plaques by promoting collagen breakdown [31]; MMP-9 is one of the biomarkers of vascular endothelial dysfunction [32], which, combined with NWI, further illustrates the vulnerability of the atheromatous plaques in the control group.

The results of previous animal studies have shown that iron overload exacerbates increased levels of blood glucose and lipids in mice [12,33]. In this study, the intraperitoneal administration of FT-H to Apo-E^(−/−)^ mice increased SF levels while decreasing blood glucose and lipid levels. In addition, the levels of MDA, a cumulative toxic product under oxidative stress conditions, were lower in the injection group than in the control group; SOD, the main substance used by the body to combat oxidative stress, was also depleted to a higher extent in the control group than in the injection group. Based on the above results, we speculate that the intraperitoneal injection of FT-H chelated free iron and reduced the exacerbation of the atherosclerotic process via iron overload, resulting in lower blood glucose, lipid and oxidative stress levels in the Apo-E^(−/−)^ mice than in the control group.

When monocytes recruit and eventually become foam cells, endothelial cells activate and secrete more chemokines and growth factors and adjacent smooth muscle cells, inducing their proliferation and the endosynthesis of extracellular matrix components such as collagen [34]. Iron overload induces apoptosis and stimulates significant MCP-1-mediated monocyte recruitment, leading to the development of atherosclerosis [12]. Ferritin has iron chelating ability and FT-H exhibits ferrous oxidase activity, which can effectively reduce iron load, and thus, reduce MCP-1-mediated monocyte recruitment, thereby decreasing endothelial cell activation and reducing the synthesis of extracellular matrix components such as collagen. Our immunohistochemical study showed that at week 20, MCP-1 was higher in the control group than in the injection group. We speculate that the iron chelation process mediated by intraperitoneal FT-H injection reduced ferroptosis and MCP-1-mediated monocyte recruitment in the arteries, which also reduced the migration of vascular smooth muscle cells to the arterial intima and reduced arterial lumen narrowing due to subintimal collagen deposition.

Previous studies on human atherosclerotic plaques have shown that the vulnerability of atherosclerotic plaques is positively correlated with the deposition of ferritin and TfR1 [35,36]. The present study showed that at week 20, the expression of FT-H, FT-L and TfR1 deposition was higher in the control group than in the injection group. Apo-E^(−/−)^ mice that underwent FT-H intraperitoneal injection did not exhibit increased FT-H deposition in their arteries, although they exhibited increased SF levels. In contrast, there was a decrease in iron metabolism protein deposition in their arteries compared to that in the control group, reducing the vulnerability of arterial plaques. In addition, the expression of NRF-2 was higher in the control group than in the injection group, which is consistent with previous studies [37], and its high expression can be observed in the advanced stages of atherosclerotic plaques. Also, the low expression of GPX-4 in the control group increased the accumulation of lipid peroxides in the aorta and aggravated the ferroptosis process in the control group.

There are several limitations to this study. First, the sample size per group was small and will be further expanded in future studies. In addition, the higher collagen content and lower expression of MMP deposition in the arteries of Apo-E^(−/−)^ mice in this study may be related to the earlier timing of our observed endpoint (Apo-E^(−/−)^ mice had not entered the fibrous atherosclerotic phase at week 20). Whether the change in collagen content in the arteries after 20 weeks, when Apo-E^(−/−)^ mice enter the fibrous atherosclerotic phase, is reduced as described in previous studies will be further investigated in subsequent studies. In addition, serum iron metabolism indicators that were not measured in this study (e.g., hepcidin, ferroportin or TfR), endothelial activation biomarkers, etc., will be included as more useful indicators in future studies.

## 5. Conclusions

The intraperitoneal administration of FT-H to Apo-E^(−/−)^ mice resulted in lower blood glucose and lipid levels; reduced iron and iron metabolism protein deposition in the aorta; reduced indices of their ferroptosis, oxidation and inflammatory aggregation; and reduced collagen deposition in the aorta, which delayed the process of aortic atherosclerosis in mice.

## Data Availability

All data generated or analyzed during this study are included in this article. Further inquiries can be directed to the corresponding author.
